# The Three Dimensional Quantitative Structure Activity Relationships (3D-QSAR) and Docking Studies of Curcumin Derivatives as Androgen Receptor Antagonists

**DOI:** 10.3390/ijms13056138

**Published:** 2012-05-18

**Authors:** Guanhong Xu, Yanyan Chu, Nan Jiang, Jing Yang, Fei Li

**Affiliations:** 1 School of Pharmacy, Nanjing Medical University, Nanjing 210029, China; E-Mails: xghchem@hotmail.com (G.X.); chuyanyan1@hotmail.com (Y.C.); jiangnan792000@163.com (N.J.); alley79@163.com (J.Y.); 2 School of Chemistry and Chemical Engineering, Nanjing University, Nanjing 210093, China

**Keywords:** CoMFA, CoMSIA, docking, androgen receptor antagonists, curcumin derivatives

## Abstract

Androgen receptor antagonists have been proved to be effective anti-prostate cancer agents. 3D-QSAR and Molecular docking methods were performed on curcumin derivatives as androgen receptor antagonists. The bioactive conformation was explored by docking the potent compound 29 into the binding site of AR. The constructed Comparative Molecular Field Analysis (CoMFA) and Comparative Similarity Indices Analysis (CoMSIA) models produced statistically significant results with the cross-validated correlation coefficients *q*^2^ of 0.658 and 0.567, non-cross-validated correlation coefficients *r*^2^ of 0.988 and 0.978, and predicted correction coefficients *r*^2^_pred_ of 0.715 and 0.793, respectively. These results ensure the CoMFA and CoMSIA models as a tool to guide the design of novel potent AR antagonists. A set of 30 new analogs were proposed by utilizing the results revealed in the present study, and were predicted with potential activities in the developed models.

## 1. Introduction

Prostate cancer is the most common malignant tumor and the second most common cause of cancer death in the male population [1]. The current treatment for prostate cancer is a combination of surgery, radiation, and chemotherapy [2]. As prostate cancer development and disease progression is hormone dependent, blockade of androgen action is the foundation of most popular therapies [3]. Castration-resistant prostate cancer (CRPC) is an advanced status of prostate cancer associated with high death rates [4]. Treatment options for CRPC are an unmet need, with current anti-androgens having been shown only to prolong survival [5]. The androgen receptor (AR) is postulated to play a key mediator of prostate cancer [6]. Over the past 2 decades, several important mechanisms of mutation in AR have been elucidated. Laboratory observations have offered clues that AR mutations have turned the growth-inhibitory effect of the current clinically used anti-androgens into a growth-promoting effect at the castration-resistant form [5,6]. This has led to an attractive strategy targeting mutant AR which offer promising potential in future treatment of CRPC.

In recent years, a number of androgen receptor antagonists have appeared, among them, Li Lin *et al*. synthesized a series of curcumin derivatives as potent selective AR antagonists. Some compounds showed significant cytotoxicity against human prostate cancer cell lines, androgen-dependent LNCaP. Anti-androgenic activity was also evaluated in LNCaP cells transfected with wild-type AR [2]. In addition, the X-ray crystal structures of AR have been determined [7] which provide useful information about the interaction with the residues near the binding site.

The three dimensional quantitative structure activity relationships (3D-QSAR) may be useful in drug discovery and design [8]. As the most popular QSAR methods, Comparative Molecular Field Analysis (CoMFA) [9] and Comparative Similarity Indices Analysis (CoMSIA) [10] studies incorporate 3D information for the ligands by searching for sites on molecules capable of being modified into better specific ligands. As a useful methodology for studying the interaction mechanism, receptor based molecular docking analysis can offer vivid interaction picture between a ligand and an acceptor [11]. Combined 3D-QSAR and docking study could offer more information to understand the structural features of bonding site of protein and the detail of protein–ligand interactions for purposive directing the design of new potential molecules [12].

In this work, QSAR and docking studies of androgen receptor antagonists with anticancer activity against human prostate cancer cell line LNCaP were carried out. An optimal 3D QSAR model for these compounds was established, and the model can be used to predict quantitatively the properties of entry antagonists not in the data set. We expect that the results can offer some reference to guide the design of novel potent AR antagonists.

## 2. Material and Methods

### 2.1. Data Sets

All curcumin derivatives and their biological activities (IC50 values) were taken from the literature [2]. In order to examine the predictive ability and robustness of the QSAR models, the test set of 7 molecules were selected randomly in such a way that the structural diversity and wide range of activity in the data set were included, and the remaining 33 compounds are treated as a training set and used to derive the 3D-QSAR models.

The structures of the compounds and their biological data are given in Table 1. The cytotoxicity bioassay was performed according to the procedures described in Lin *et al*. [2]. IC50 values are mean concentrations that inhibit growth by 50% and variation between replicates was less than 5%. The IC50 values in units of μM were transformed in pIC50 (-log IC50) in order to give numerically larger data values.

### 2.2. Molecular Modeling and Alignment

All molecular modeling and 3D-QSAR calculations were done using SYBYL X 1.3 (Tripos Associates Inc., St. Louis, Missouri, USA, 2011). Molecular building was done with a molecule sketch program on the same software. The molecular geometry of each compound was first minimized using a standard Tripos molecular mechanics force field with 0.01 kcal/(mol Å) energy gradient convergence criterion. Partial atomic charges were calculated by the Gasteiger-Hückel method and energy minimizations were performed using the Powell method 1000 iterations [13].

**Figure 1:**
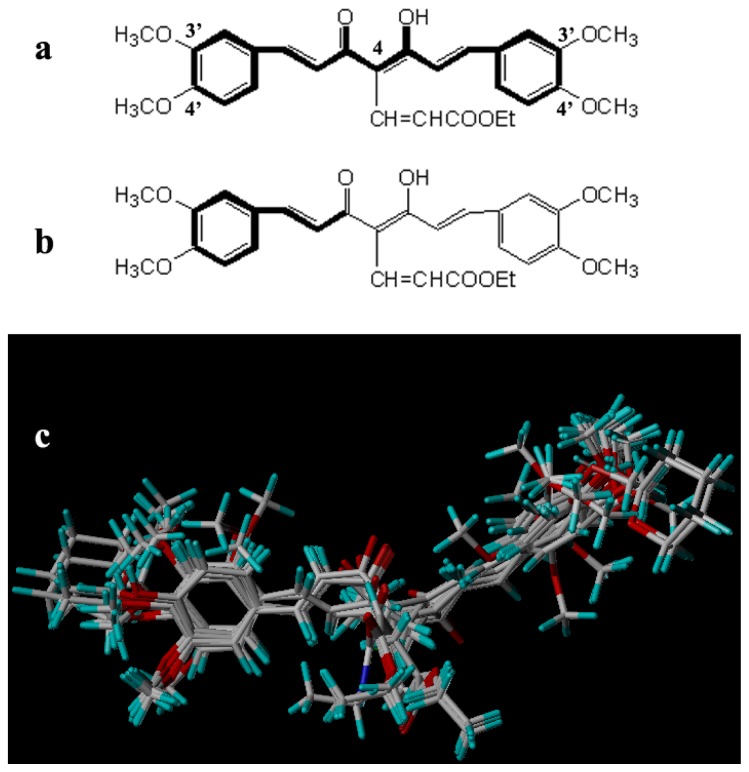
Molecular alignment of the compounds in the training set.

The accuracy of the prediction of QSAR model and reliability of the contour maps are directly dependent on the structural alignment rule [14]. In order to obtain the best possible 3D-QSAR statistical model, two different alignment rules were adopted. During the process, the lowest energy conformation of compound 29 was used as the template for the alignment, because it is one of the most active compounds in Table 1. Figure 1a describes the common substructure for the alignment which is marked in bold. However, due to with no such substructure of Figure 1a in the structures, compounds 1–10, 28 and 31 were aligned based on another common substructure depicted in bold as shown in Figure 1b,c shows the resulting ligand-based alignment model.

### 2.3. CoMFA and CoMSIA Field Calculation

The standard CoMFA procedure as implemented by SYBYL X 1.3 was performed. For each aligned sets of molecules were positioned inside a 3D cubic lattice with a grid spacing of 2.0 Å (default distance) in all Cartesian directions was generated to enclose the molecule aggregate. A sp^3^ carbon atom with a charge of +1.0 and a van der Waals radius of 1.52 Å was used as a probe; this atom was placed at every lattice point to calculate various steric and electrostatic fields by the CoMFA standard method with default cut-off energy of 30.0 kcal/mol [15]. In order to reduce noise and improve efficiency, column filtering was set to 1.0 kcal/mol. The fields generated were scaled by CoMFA standard in SYBYL automatically.

The CoMFA region focusing is the application of weights to the lattice points in a CoMFA region to enhance or attenuate the contribution of those points to subsequent analyses. When the weights are StDev*Coefficient values, the process is exactly equivalent to image enhancement of the derived CoMFA maps for getting the better models [16].

The CoMSIA method, discovered by Klebe [10], has advantages over CoMFA technique such as greater robustness regarding both region shifts and small shifts within the alignments [16]. With the standard parameters and no arbitrary cutoff limits, five fields associated, namely, steric, electrostatic, hydrophobic, hydrogen bond donor and hydrogen bond acceptor, were calculated using the same lattice box created for CoMFA. The default value of 0.3 was used as the attenuation factor.

### 2.4. Partial Least Square Analysis

The partial least squares (PLS) methodology was used to derive a linear relationship for the 3D-QSAR, and cross-validation was performed using the leave-one-out(LOO) method [17] to choose the optimum number of components (ONC) and assess the statistical significance of each model. In PLS, the independent variables were the CoMFA and CoMSIA descriptors, and pIC_50_ values were used as dependent variables [16]. The ONC was the number of components that led to the highest cross-validated correlated correlation coefficient *q*^2^ (or *r*^2^_cv_). Before the PLS analysis, the CoMFA and CoMSIA columns were filtered by using column filtering. Non-cross-validation was performed to calculate conventional *r*^2^_ncv_ using the same number of components. To further assess the robustness and statistical confidence of the derived models, bootstrapping analysis for 100 runs was performed [9,18].

### 2.5. Molecular Docking

To determine the probable binding conformations and offer more insight into the understanding of the interactions of androgen receptor antagonists, molecular docking analysis was carried out using the Surflex Dock in SYBYL. The crystal structure of AR was retrieved from RCSB Protein Data Bank (PDB entry code: 1T65) [7]. The protein structures were utilized in subsequent docking experiments without energy minimization. All ligands and water molecules have been removed at first, the polar hydrogen atoms and AMBER7FF99 charges were added. Protomol, a computational representation of the intended binding site, is used to guide molecular docking [19]. Jinming Z. *et al*. predicted binding mode of AR antagonists in the antagonistic model of wild type AR ligand-binding domain (WT AR-LBD). E709, Q738, W741, M742, L880, L881, and V889 were key residues of the active site to form hydrogen bonds or a hydrophobic pocket [6]. Therefore, the active sites were considered to be the potential receptor’s binding sites. In view of this, residues mode was adopted to generate the protomol by specifying residues in the receptor near Helix 12 in this study. The protomol bloat value was set as 1 and the protomol threshold value as 0.5 when a reasonable binding pocket was obtained. Other parameters are established by default in software.

## 3. Results and Discussion

### 3.1. CoMFA and CoMFA Region Focusing

**Figure 2:**
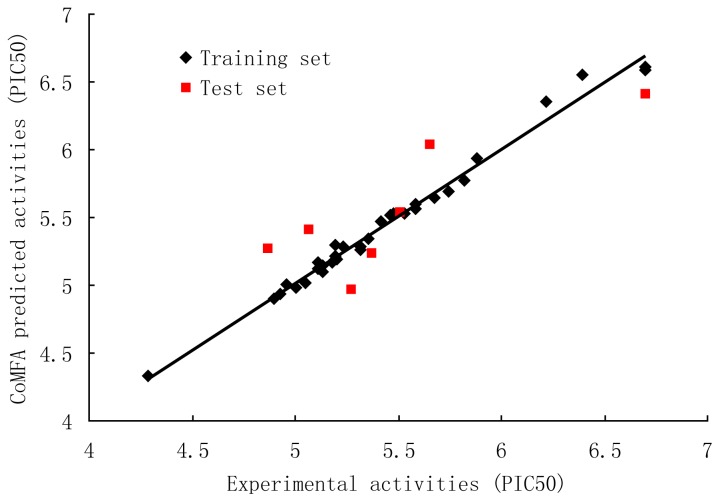
Correlation between the experimental and CoMFA (region focusing) predicted activities of compounds.

The results of CoMFA studies are summarized in Table 2. The optimal number of components was determined by selecting highest *q*^2^ value. PLS analysis showed a high *q*^2^ value of 0.564 with 6 components for CoMFA. The non-cross-validated PLS analysis results in a conventional *r*^2^_ncv_ of 0.986; F is 304.611, and a standard error of estimation (SEE) of 0.068. When these fields were focused, the *q*^2^ improved and produced highest *q*^2^ of 0.658 with 6 components, *F* = 352.278, *r*^2^_ncv_ = 0.989 and SEE = 0.063. The steric and electrostatic contributions were 48.8% and 51.2%, respectively. Bootstrap analysis for 100 runs was then carried out for further validation of the model by statistical sampling of the original data set to create new data sets. The higher r^2^ bootstrap value 0.992 for CoMFA with standard error value of 0.049 is supporting the statistical validity of the developed models. The predicted activities for the antagonists *versus* their experimental activities are listed in Table 3 and the correlation between the predicted activities and the experimental activities is depicted in Figure 2. The predictive correlation coefficient *r*^2^_pred_ was found to be 0.715 for the test set. Statistical results suggest that the CoMFA model is a reliable predictor.

### 3.2. CoMSIA

**Figure 3:**
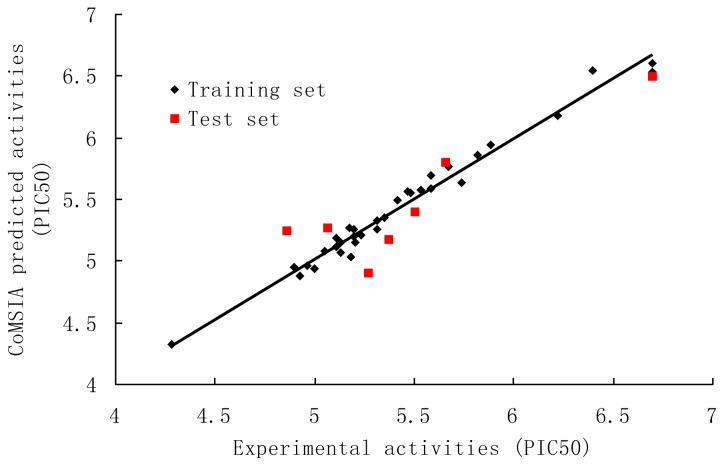
Correlation between the experimental and CoMSIA predicted activities of compounds.

The PLS results of CoMSIA analysis using different combinations were depicted in Table 4. The SEHD field descriptors exhibited highest *q*^2^, better SEE and *F* values than the others. Therefore, the combination of steric (S), electrostatic (E), hydrophobic (H) and hydrogen bond donor (D) fields was selected as the best model. The CoMSIA model gave a *q*^2^ of 0.567 with an optimized component number of 5. A high *r*^2^_ncv_ of 0.978 with a low SEE of 0.083 and *F* value of 241.534. High values of the electrostatic (43.0%) and hydrogen bond donor (23.6%) fields show the importance of the electrostatic and hydrogen bond donor nature of the substituents on the core. The other descriptors, steric (16.0%) and the hydrophobic (17.3%) also have contribution. The predicted activities for the antagonists *versus* their experimental activities are listed in Table 3 and the correlation between the predicted activities and the experimental activities is depicted in Figure 3. The predictive correlation coefficient *r*^2^_pred_ was found to be 0.793 for the test set. Bootstrap analysis for 100 runs was then carried out for further validation of the model by statistical sampling of the original data set to create new data set. This *r*^2^ of bootstrap value is 0.983 for CoMSIA with standard error value of 0.069, supporting further the statistical validity of the developed models. All the results indicate that the CoMSIA model is also fairly predictive.

### 3.3. Contour Maps Analysis

The best CoMFA and CoMSIA models are selected to construct the stdev*coeff contour maps to view the field effects on the target features. All the contours represented the default 80% and 20% level contributions for favorable and unfavorable regions, respectively, except 70% and 30% level contributions in figure of hydrogen bond donor contour maps. The maps showed regions where differences in molecular fields are associated with differences in biological activity.

#### 3.3.1. CoMFA Contour Maps

**Figure 4:**
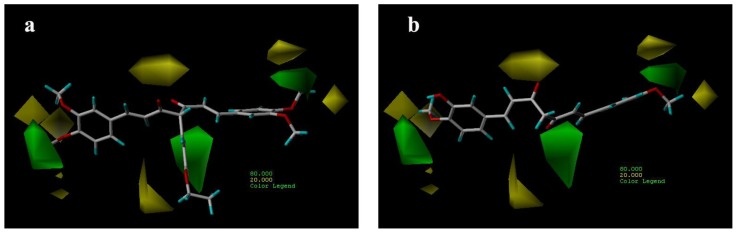
CoMFA steric contour maps for compounds: (**a**) compound 29; (**b**) compound 15.

CoMFA steric contour maps are shown in Figure 4. The steric interaction is represented by green and yellow contours, while electrostatic interaction is denoted by red and blue contours. A large green contour was found near the substituent group of C-4 position indicating that bulky substituents were preferred in this region (Figure 4a). This may be the reason why compounds with alkyl substituents in this area, e.g., compounds 32, 33 and 40, are more potent AR antagonist activity than molecules without any substituent at this particular position, such as compounds 14, 15 (Figure 4a) and 22.

**Figure 5:**
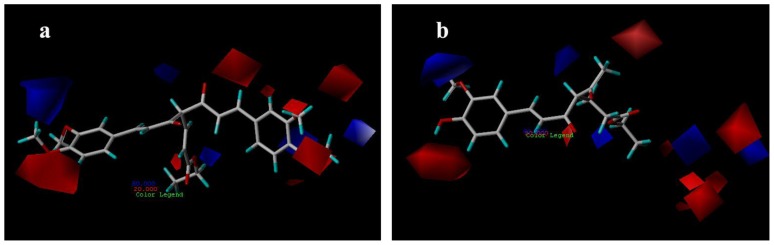
CoMFA electrostatic contour maps for compounds: (**a**) compound 29; (**b**) compound 7.

The CoMFA electrostatic contour plots for compounds are displayed in Figure 5. The blue contours indicate that electropositive substituents would increase the AR antagonist activity with protein, while red color indicates that they should be the electron rich groups [18]. Since the red contours were found near the methoxyl group of compounds 29, which is an electron rich functionality, compounds 29 exhibit high AR antagonist activity (Figure 5a). A large blue contour was found near the methoxyl substituent on phenyl ring of compound 7 (Figure 5b), indicating that negatively charged groups are disfavored at this position, and that is a possible reason why compound 7 displays less potent AR antagonist activity than compound 29.

#### 3.3.2. CoMSIA Contour Maps

**Figure 6:**
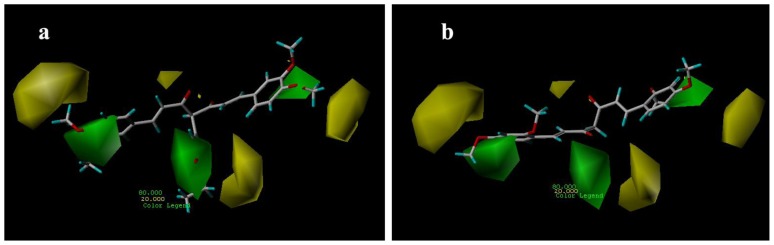
CoMSIA steric contour maps for compounds: (**a**) compound 29; (**b**) compound 15.

**Figure 7:**
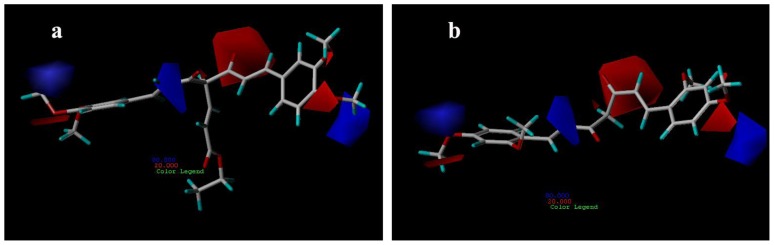
CoMSIA electrostatic contour maps for compounds: (**a**) compound 29; (**b**) compound 15.

**Figure 8:**
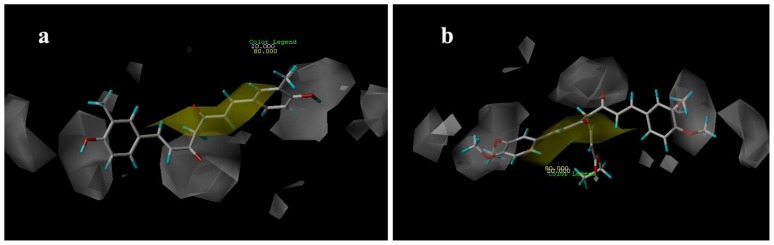
CoMSIA hydrophobic contour maps for compounds: (**a**) compound 21; (**b**) compound 29.

**Figure 9:**
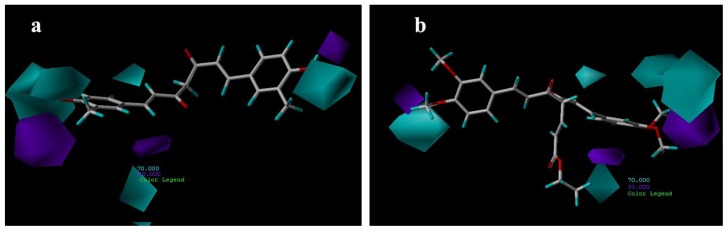
CoMSIA hydrogen bond donor contour maps for: (**a**) compound 21; (**b**) compound 29.

The CoMSIA contour maps, derived using steric, electrostatic, hydrophobic and hydrogen bond donor fields, are represented in Figures 6–9. CoMSIA steric and electrostatic contours are more or less similar to those of the CoMFA. As in case of CoMFA, a large green contour was found overlapping the substituent group of C-4 position (Figure 6a), to indicate that bulky substituents were preferred in this region compared with compound 15 (Figure 6b).

Figure 7 shows the CoMSIA electrostatic fields denoted by red and blue contours. Red contours represent regions where negatively charged substituents are preferred on ligands and blue contours indicate regions where electron-rich substituents are unfavorable for the activity. The methoxyl groups of compound 29 are all near the red areas (Figure 7a), the favored position for electronegative groups. While one the methoxyl groups of compounds 15 is near the blue contour, which means that this group is not favored in this region and will lead to a decrease in the AR antagonist activity (Figure 7b).

Figure 8 shows the hydrophobic contour maps in which yellow and gray contours indicate the regions where hydrophobic and hydrophilic groups are favored by the model, respectively. A yellow contour overlapping the linker including aliphatic hydrocarbon structure of compound 21 group indicates that hydrophobic substituent at this position would increase the AR antagonist activity (Figure 8a). The two large gray contours near the hydroxyl groups indicate that hydrophilic groups at these positions are favorable. These results are quite similar to those of compound 29 (Figure 8b).

Hydrogen-bond donor contour maps from CoMSIA are shown in Figure 9. Here, the maps generated depict regions having scaled coefficients 70% (favored) or 30% (disfavored). The cyan contours represent the regions where hydrogen bond-donating groups increase the activity; the purple contours represent the regions where hydrogen bond-donating groups decrease the activity. As shown in Figure 9a, the cyan contours are near the H-bond donor, hydroxyl groups, of compound 21, whereas the methoxy groups of compound 29 are present near the purple contour (Figure 9b) as H-bond acceptors.

### 3.4. Docking Analysis

**Figure 10:**
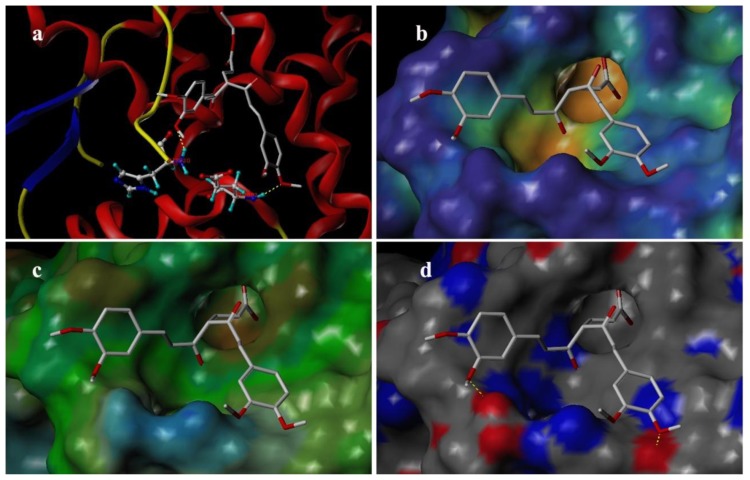
Binding conformations of the compound 29 at the bonding site of androgen receptor (AR).

Docking was employed to explore the binding mode between these curcumin derivatives and the AR, to examine the stability of 3D-QSAR models previously established. We selected the most potent antagonist 29 in the experiment to perform the deeper docking study and discussion below. In order to visualize secondary structure elements, the MOLCAD program was applied. Figure 10a showed the secondary structure of the receptor. The key residues and hydrogen bonds were labeled. As shown in Figure 10a, the oxygen atom of methoxy group acted as a hydrogen bond acceptor by forming two H-bonds with the –NH_2_ group of the HIS920 residue and the –NH– group of the GLU893, respectively. The observations taken from Figure 10 were in agreement with the corresponding CoMSIA hydrogen bond contour maps.

Figure 10b depicted the MOLCAD cavity depth potential surfaces structure of the binding site within the compound 29. The cavity depth color ramp ranges from blue (low depth values = outside of the pocket) to light red (high depth values = cavities deep inside the pocket) [20]. The substituent group of C-4 position of compound 29 was oriented in a light red region which demonstrated that this part was anchored deep inside the pocket. The remaining parts of compound 29 are found in the cyan area which indicated that the benzene ring was anchored outside the pocket.

Figure 10c showed the MOLCAD lipophilic potential (LP) surface of the binding area, the color for LP ranges from brown (highest lipophilic area of the surface) to blue (highest hydrophilic area). The linker of molecule was oriented to a brown region, suggesting that a hydrophobic substituent may be favored; the methoxy group was oriented to the blue and white areas which indicated that a hydrophilic group would be favorable. The observations taken from Figure 10c satisfactorily matched those of the CoMSIA hydrophobic contour map.

Figure 10d displayed the MOLCAD hydrogen bonding sites of the binding surfaces, ligands can be docked to proteins by matching the patterns displayed on the surface, the red is hydrogen donors and the blue is hydrogen acceptors. As shown in Figure 10d, the methoxy groups were oriented to a red surface, which indicated that the surfaces of this site were hydrogen bond donors, and a hydrogen bond acceptor substituent would be favorable. The observations taken from this hydrogen bonding sites satisfactorily matched the corresponding CoMSIA hydrogen bond contour maps.

### 3.5. Summary of Structure-Activity Relationship

**Figure 11:**
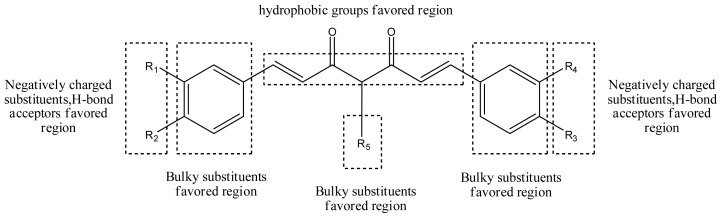
Summary of structure-activity relationship.

The structure-activity relationship revealed by 3D-QSAR and molecular docking studies were illustrated in Figure 11. The negatively charged substituents, H-bond acceptors at R1, R2, R3, and R4 position would increase the activity; the substitution at the C-4 position of the linker is very crucial for improved activity in this compound class. The hydrophobic substituent at the position of the linker would increase the activity. Biphenyl rings at the both two sides of curcumin derivatives are required for the cytotoxic same as those in bicalutamide, a known and clinically used AR antagonist. Here, the hydrophobic property of benzene ring plays a key role in the anti-androgenic activities.

### 3.6. Designs for New Molecules

**Figure 12:**
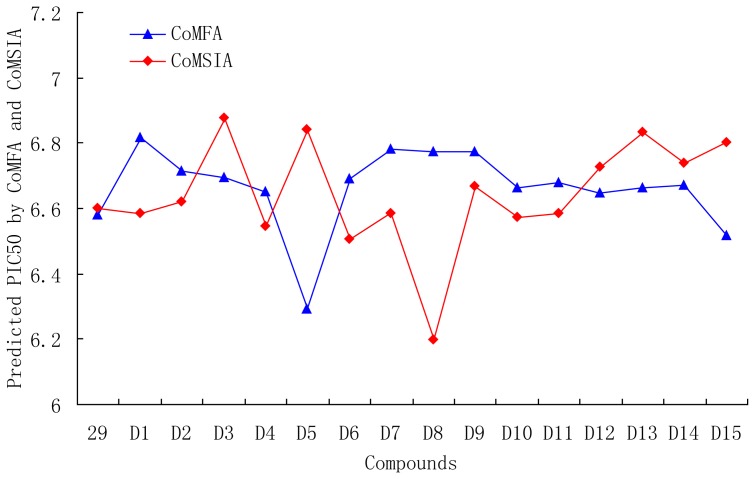

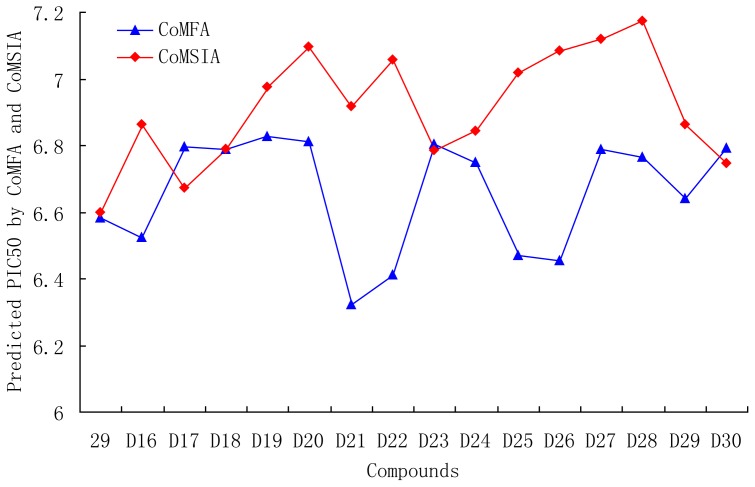
Graph of the predicted pIC50 of the designed molecules *versus* compound 29.

Based on QSAR and docking results, antagonists 29, with the highest activity, was taken as a template to design new compounds. A set of 30 new compounds with high predicted activity were designed and assessed (Table 5), these molecules were aligned to the database and their activities were predicted by the CoMFA and CoMSIA models previously established. The chemical structures and predicted pIC50 values of these compounds were shown in Table 5, and the graph of their predicted pIC50 values *versus* the most active compound 29 was shown in Figure 12. Most of the designed molecules exhibited better predicted pIC50 values than compound 29 in CoMFA or CoMSIA models. Molecules D2, D3, D7, D9–14, D17, D19–20, D23–24, and D27–30 displayed significantly improved predicted activities than compound 29 in both the CoMFA and CoMSIA models. The results validated the structure activity relationship obtained by this study.

## 4. Conclusion

In the present study, 3D-QSAR analyses have been applied to a set of curcumin derivatives. The models have proven to be statistically robust with higher *q*^2^ and *r*^2^. Also, as demonstrated in our study, 3D-QSAR and docking methods were employed to understand the structural features responsible for the affinity of the ligands for AR. These results provided crucial clues that were used to design novel androgen receptor antagonists with high predicted potent activity. A set of 30 novel derivatives were designed by utilizing the structure-activity relationship taken from the present study.

## Figures and Tables

**Table 1:** Structures and experimental anticancer activities (against human prostate cancer cell line LNCaP) of the curcumin derivatives.

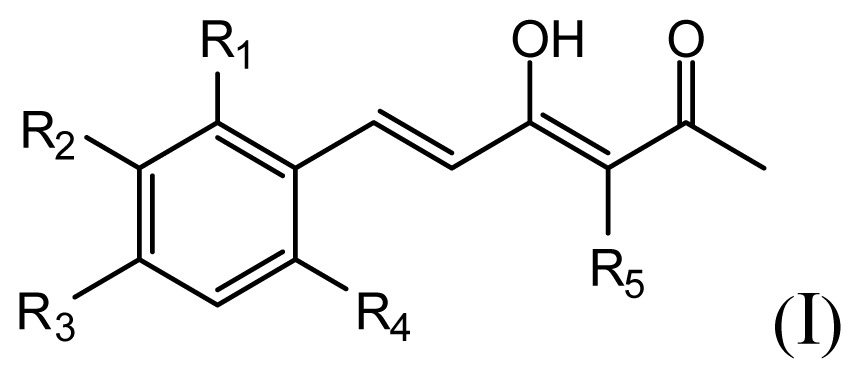							

Compound	R_1_	R_2_	R_3_	R_4_	R_5_	IC_50_ (μM)	pIC_50_
1	H	OMe	OH	H	H	6.2	5.208
2	H	OMe	OMe	H	H	6.6	5.180
3 *	H	OH	OMe	H	H	5.3	5.276
4	OMe	H	OMe	H	H	9.9	5.004
5	OMe	OMe	OMe	H	H	5.8	5.237
6	H	OMe	OMe	OMe	H	12.5	4.903
7	H	OMe	OH	H	(CH_2_)_2_COOEt	51.5	4.288

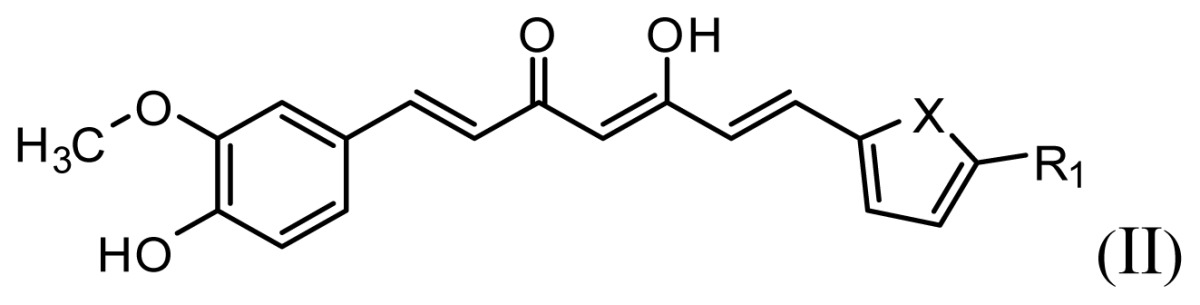							

**Compound**		**R_1_**		**X**		**IC_50_** **(μM)**	**pIC_50_**

8		CH_2_OH		O		7.3	5.137
9		H		S		6.3	5.201
10*		H		NH		13.6	4.866

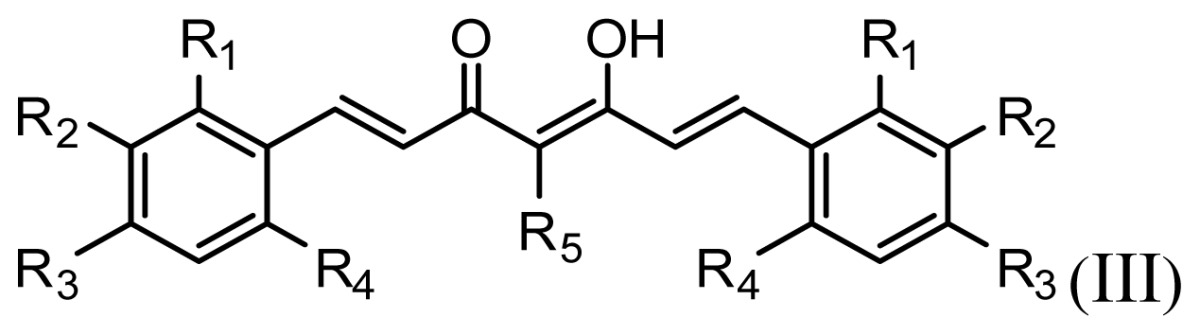							

**Compound**	**R_1_**	**R_2_**	**R_3_**	**R_4_**	**R_5_**	**IC_50_** **(μM)**	**pIC_50_**

11	H	OMe	OH	H	H	3.8	5.420
12	H	OMe	OMe	H	H	1.3	5.886
13	H	OMe	OH	H	(CH_2_)_2_COOEt	1.5	5.824
14	H	OH	OMe	H	H	10.9	4.963
15	OMe	H	OMe	H	H	11.8	4.928
16	OMe	OMe	OMe	H	H	4.8	5.319
17	H	OMe	OMe	OMe	H	2.9	5.538
18*	H	OMe	OTHP	H	(CH_2_)_2_COOEt	4.2	5.377
19	H	OMe	OEt	H	H	6.5	5.187
20	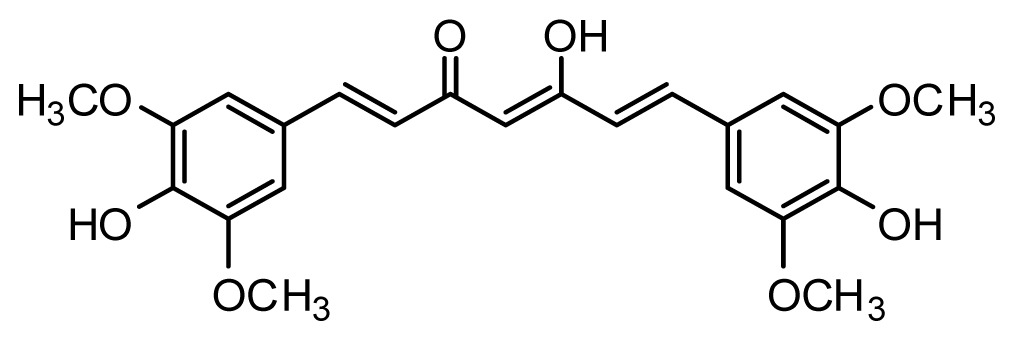					2.6	5.585
21	H	Me	OH	H	H	1.8	5.745
22	H	Me	OMe	H	H	7.7	5.114

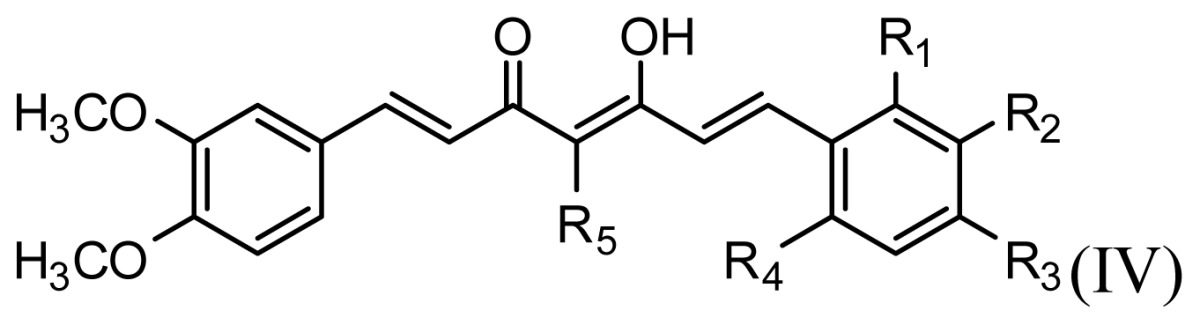							

**Compound**	**R_1_**	**R_2_**	**R_3_**	**R_4_**	**R_5_**	**IC_50_** **(μM)**	**pIC_50_**

23	H	OMe	OH	H	H	3.3	5.481
24	H	OH	OMe	H	H	4.8	5.319
25	OMe	OMe	OMe	H	H	7.7	5.114
26*	H	OMe	OMe	OMe	H	8.6	5.066
27	H	OMe	OH	H	(CH_2_)_2_COOEt	2.1	5.678

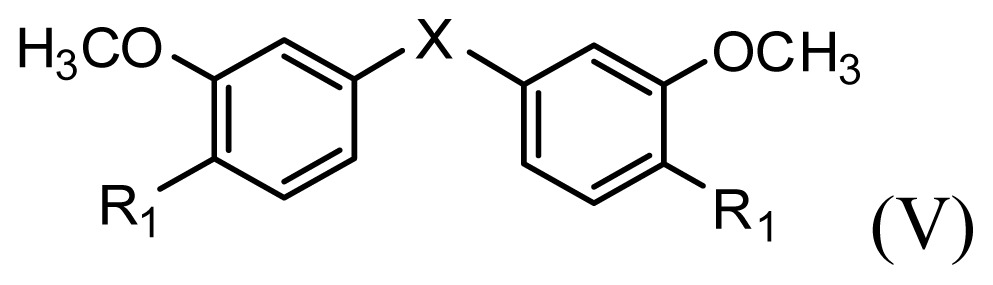							

**Compound**	**R_1_**		**X**			**IC_50_** **(μM)**	**pIC_50_**

28 *	OH		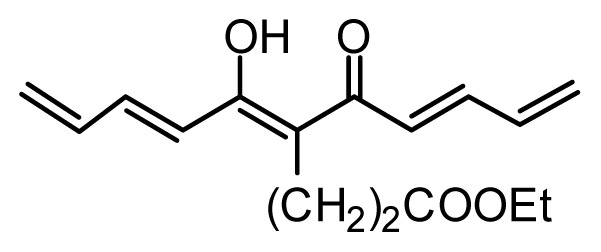			3.1	5.509
29	OMe		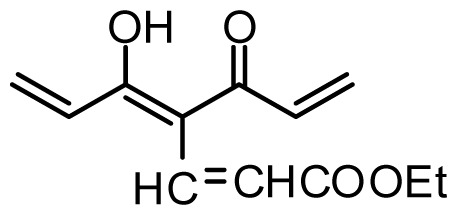			0.2	6.699
30	OTHP		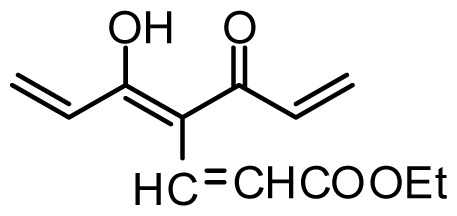			2.6	5.585
31 *	OMe		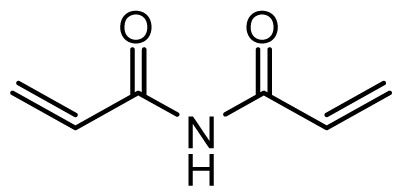			2.2	5.658
32	OMe		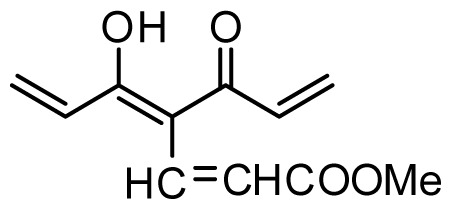			0.4	6.398
33	OMe		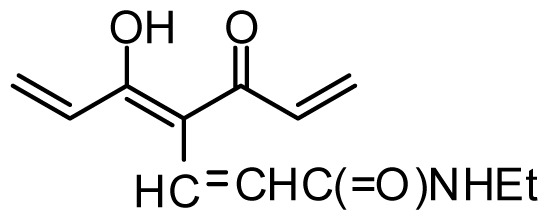			0.6	6.222
34 *	OMe		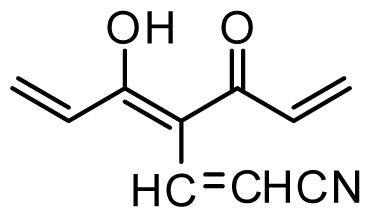			0.2	6.699
35	OH		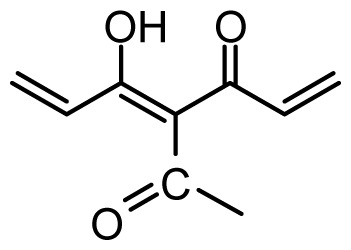			8.8	5.056
36	OTHP		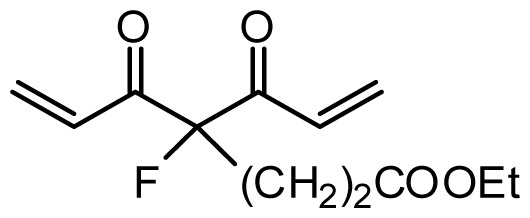			7.3	5.137
37	OH		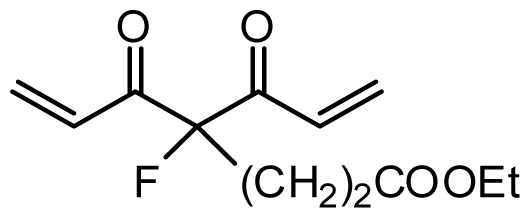			6.3	5.201
38	OMe		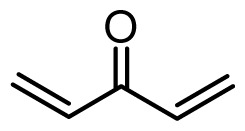			3.4	5.469
39	OH		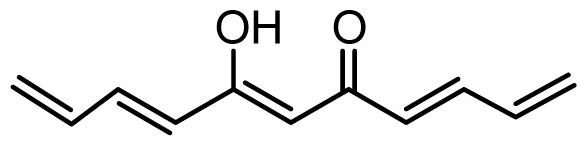			4.4	5.357
40	OMe		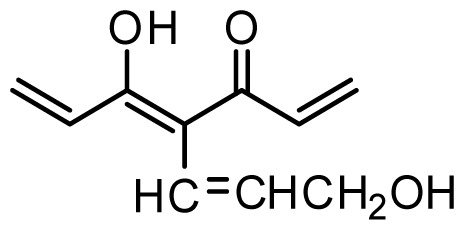			0.2	6.699

* Compounds taken for the test set.

**Table 2:** Statistical quality parameters of different molecular interaction field methods.

Component a	*q*^2^^b^	*r*^2^_ncv_ c	*F*^d^	SEE^e^
A–CoMFA region focusing model in Gs = 1 in various numbers of components f				
1	0.306	0.533	35.367	0.357
2	0.396	0.738	42.156	0.272
3	0.501	0.893	80.916	0.176
4	0.590	0.955	149.067	0.116
5	0.629	0.978	237.429	0.083
**6**	**0.658**	**0.988**	**352.278**	**0.063**
B–CoMFA model in Gs = 2 in various numbers of components f				
1	0.209	0.395	23.191	0.395
2	0.273	0.688	33.010	0.297
3	0.399	0.881	71.577	0.186
4	0.432	0.940	109.490	0.135
5	0.526	0.976	223.159	0.086
6	0.564	0.986	304.611 0.068	

a Optimum number of components (ONC) obtained from cross-validated PLS analysis and same used in final non-cross-validated analysis;

b *q*^2^: Cross-validated correlation coefficient;

c *r*^2^_ncv_: Non-cross-validated correlation coefficient;

d *F*: F-test value;

e SEE: Standard error of estimate;

f Column filtering = 1.0 kcal/mol.

**Table 3:** Comparative Molecular Field Analysis (CoMFA)/Comparative Similarity Indices Analysis (CoMSIA) predicted activity (PIC50) of compounds.

Compound	Predicted Activity (CoMFA)	Predicted Activity (CoMSIA)
1	5.191	5.124
2	5.163	5.257
3*	4.963	4.896
4	4.971	4.935
5	5.277	5.202
6	4.900	4.964
7	4.323	4.333
8	5.145	5.144
9	5.208	5.242
10 *	5.264	5.243
11	5.463	5.495
12	5.929	5.925
13	5.763	5.850
14	5.003	4.960
15	4.929	4.879
16	5.281	5.325
17	5.522	5.567
18 *	5.235	5.175
19	5.167	5.020
20	5.589	5.586
21	5.684	5.634
22	5.115	5.187
23	5.526	5.554
24	5.255	5.249
25	5.157	5.115
26 *	5.412	5.260
27	5.642	5.747
28 *	5.540	5.389
29	6.580	6.606
30	5.559	5.697
31 *	6.039	5.800
32	6.543	6.543
33	6.347	6.169
34 *	6.407	6.487
35	5.013	5.072
36	5.094	5.062
37	5.290	5.186
38	5.507	5.567
39	5.337	5.353
40	6.603	6.529

* Compounds taken for the test set.

**Table 4:** Regression summary of CoMFA and CoMSIA models.

							Field Contribution in %				
Descriptors	ONC	*q*^2^	*r*^2^_ncv_	*r*^2^_pred_ a	SEE	*F*	S	E	H	D	A
CoMFA											
SE	6	0.658	0.988	0.715	0.063	352.278	48.8	51.2	–	–	–
CoMSIA b											
SE	5	0.498	0.967		0.102	156.626	27.2	72.8	–	–	–
SHE	6	0.536	0.985		0.069	292.088	19.6	57.6	22.7	–	–
SED	6	0.519	0.983		0.075	247.310	22.3	52.0	–	25.7	–
SEA	5	0.404	0.965		0.105	148.070	19.7	55.0	–	–	25.3
**SEHD**	**5**	**0.567**	**0.978**	**0.793**	**0.083**	**241.534**	**16.0**	**43.0**	**17.3**	**23.6**	–
SEDA	5	0.486	0.969		0.098	170.503	15.6	38.9	–	23.5	21.9
SEHA	6	0.426	0.983		0.074	255.498	15.3	45.9	17.8	–	20.9
SEHDA	6	0.514	0.983		0.074	250.217	12.7	33.4	14.3	21.6	18.0

a *r*^2^_pred_: Predictive *r*^2^;

b Field contributions: Steric (S) and electrostatic (E) field from CoMFA; Steric (S), electrostatic (E), hydrophobic (H), donor (D), and acceptor (A) fields from CoMSIA.

**Table 5:** The structures and predicted pIC50 values of newly designed derivatives.

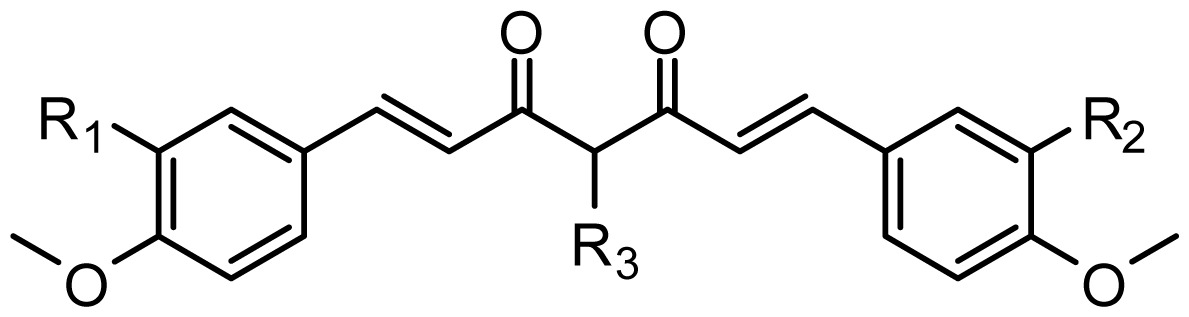					
Compound ID	Substituent			Predicted pIC50	

	R_1_	R_2_	R_3_	COMFA	COMSIA
29	OMe	OMe	CH=CHCOOEt	6.582	6.599
D1	CN	CN	CH=CHCOOEt	6.817	6.583
D2	SO_3_H	SO_3_H	CH=CHCOOEt	6.714	6.619
D3	NO_2_	NO_2_	CH=CHCOOEt	6.696	6.876
D4	CF_3_	CF_3_	CH=CHCOOEt	6.651	6.544
D5	COOH	COOH	CH=CHCOOEt	6.293	6.840
D6	CHO	CHO	CH=CHCOOEt	6.691	6.506
D7	Br	Br	CH=CHCOOEt	6.783	6.583
D8	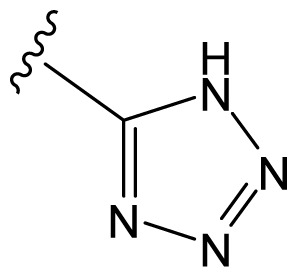	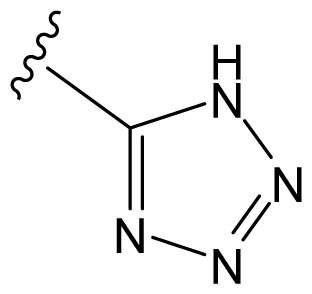	CH=CHCOOEt	6.773	6.196
D9	NO_2_	CN	CH=CHCOOEt	6.774	6.666
D10	B(OH)_2_	B(OH)_2_	CH=CHCOOEt	6.664	6.571
D11	CN	CN	CH=CH(CH_2_)_3_CH_3_	6.680	6.585
D12	OMe	OMe	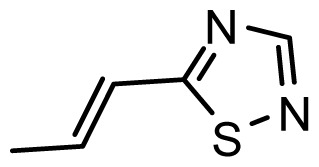	6.648	6.727
D13	OMe	OMe	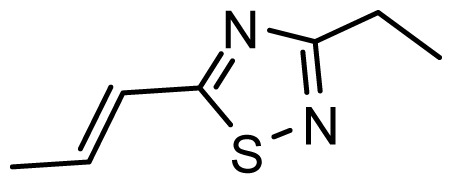	6.662	6.832
D14	OMe	OMe	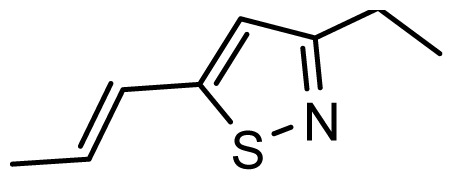	6.670	6.740
D15	OMe	OMe	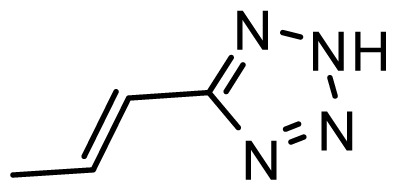	6.518	6.802
D16	OMe	OMe	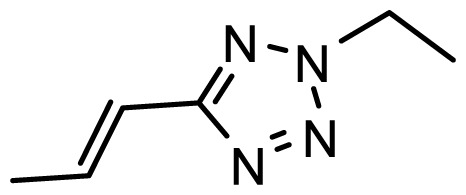	6.526	6.864
D17	CN	CN	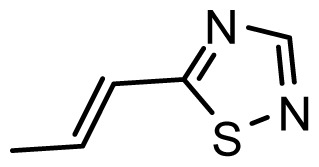	6.798	6.670
D18	CN	CN	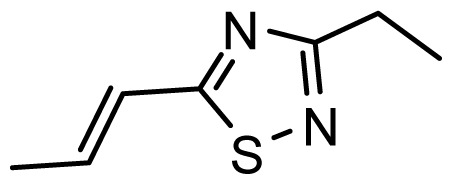	6.787	6.787
D19	NO_2_	NO_2_	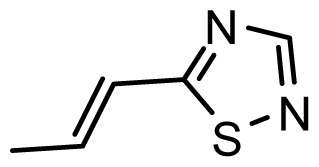	6.828	6.973
D20	NO_2_	NO_2_	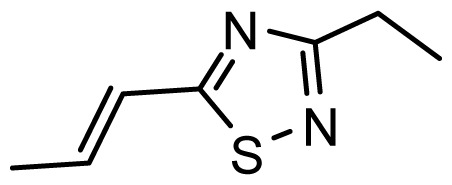	6.813	7.094
D21	COOH	COOH	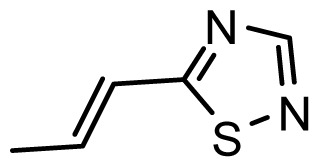	6.020	6.917
D22	COOH	COOH	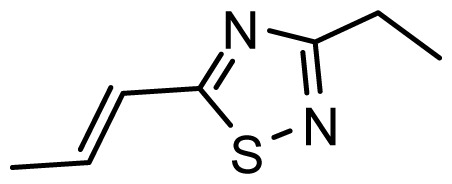	6.112	7.058
D23	CN	CN	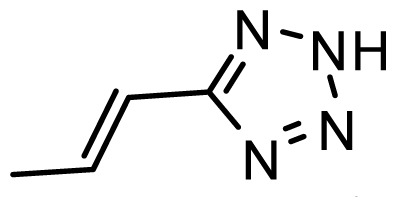	6.804	6.783
D24	CN	CN	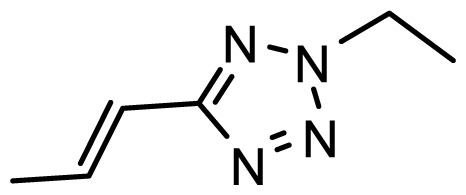	6.749	6.842
D25	COOH	COOH	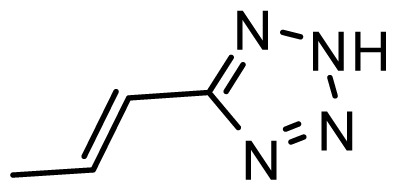	6.068	7.017
D26	COOH	COOH	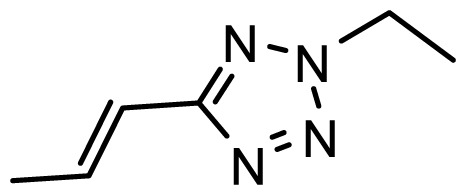	6.053	7.084
D27	NO_2_	NO_2_	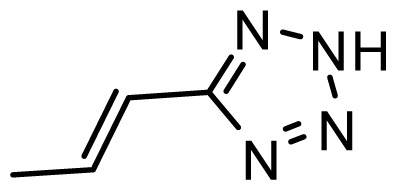	6.789	7.119
D28	NO_2_	NO_2_	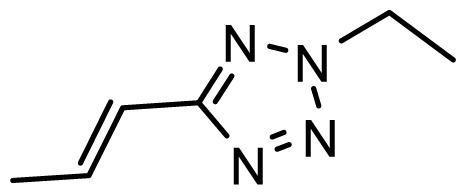	6.765	7.172
D29	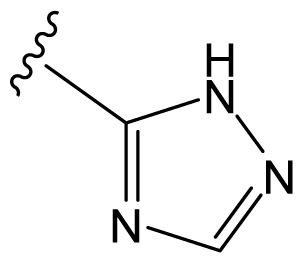	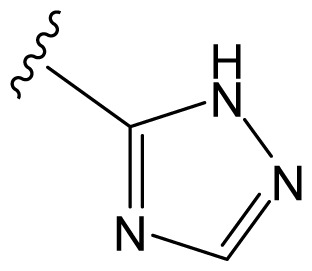	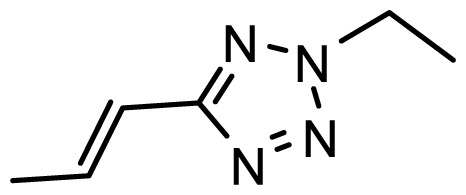	6.641	6.863
D30	Br	Br	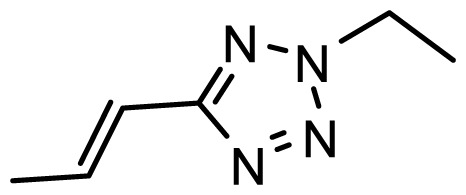	6.794	6.746
